# Assessing the Perception of the Family Adoption Program Among Medical Students, Adopted Families, and Medical Faculty: A Mixed-Methods Study

**DOI:** 10.7759/cureus.110188

**Published:** 2026-06-03

**Authors:** Shefali Jain, Ambrish Mishra, Pushpendra Sahu, Aastha Kalra, Priyesh Marskole

**Affiliations:** 1 Community Medicine, Government Medical College, Satna, Satna, IND; 2 Preventive Medicine, Rabindranath Tagore (RNT) Medical College, Udaipur, IND; 3 Community Medicine, Government Medical College, Seoni, Seoni, IND

**Keywords:** clinical skills, community healthcare, experiential learning, family adoption program, medical education

## Abstract

Background

The Family Adoption Program (FAP) is a community-oriented medical education initiative in which medical students adopt families to provide basic healthcare support while enhancing their clinical, communication, and observational skills. Understanding the perspectives of medical students, adopted families, and faculty members is essential for evaluating the program’s educational and community impact and identifying areas for improvement.

Methods

A mixed-methods study was conducted among 220 participants, including 150 second-year medical students, 50 adopted family members, and 20 faculty members involved in the FAP. Quantitative data were collected from students using a structured 5-point Likert-scale questionnaire assessing perceived effectiveness, learning experiences, and challenges associated with the program. Qualitative data were obtained through in-depth interviews with faculty members and focus group discussions with adopted family members. Quantitative data were analyzed using descriptive and inferential statistics, while qualitative data were analyzed using Braun and Clarke’s thematic analysis framework.

Results

Most students reported that the FAP improved their communication skills (85.3%) and clinical understanding (81.3%), while 78.0% perceived enhanced confidence in community interaction and patient counseling. Faculty members emphasized the program’s role in bridging theoretical learning with real-world healthcare exposure. Adopted families reported increased health awareness, improved healthcare-seeking behavior, and better understanding of preventive practices. Despite these benefits, students identified major challenges, including academic workload (72.0%), transportation difficulties (64.7%), and scheduling conflicts (61.3%). Families also expressed concerns related to communication barriers, trust-building, and difficulty adhering to medical advice. Qualitative analysis highlighted the need for improved orientation sessions, stronger faculty mentorship, structured follow-up mechanisms, and enhanced community engagement.

Conclusion

The FAP is an effective experiential learning strategy that strengthens medical students’ clinical exposure, communication abilities, and understanding of community healthcare while simultaneously improving health awareness among adopted families. However, logistical barriers, academic burden, and communication challenges may limit optimal implementation. Strengthening institutional support, structured mentorship, and flexible scheduling can improve the long-term sustainability and educational impact of the program.

## Introduction

The Family Adoption Program (FAP), introduced by the National Medical Commission, is an innovative initiative in undergraduate medical education aimed at strengthening community-oriented learning and promoting holistic healthcare delivery [1]. The program assigns medical students to families within rural or urban communities, facilitating longitudinal engagement, understanding of health-related behaviors, and provision of basic health education.

Competency-based medical education (CBME) emphasizes the development of practical skills, communication abilities, and patient-centered care through early clinical and community exposure [2]. Community-based learning experiences enable students to understand real-world healthcare delivery and enhance their ability to integrate theoretical knowledge with clinical practice [3]. Furthermore, exposure to families and communities helps students recognize the importance of social determinants of health, which play a crucial role in influencing health outcomes and healthcare access [4].

The FAP aligns closely with these principles by promoting experiential learning and sustained interaction with communities. Experiential learning theory suggests that knowledge is constructed through direct experience and reflection, making community-based programs particularly valuable in medical training [5]. Previous studies have demonstrated that participation in such programs improves students’ communication skills, clinical reasoning, empathy, and understanding of community health needs [6,7].

In addition, structured communication training has been shown to enhance doctor-patient interactions and improve the effectiveness of healthcare delivery, highlighting the importance of communication skill development in medical curricula [8]. Community-based programs also contribute to building trust between healthcare providers and the community, which is essential for effective healthcare delivery and improved health-seeking behavior [9].

Despite these advantages, several challenges have been reported in the implementation of such programs, including logistical constraints, academic workload, transportation difficulties, and communication barriers [6,10]. Furthermore, the success of these programs depends on effective supervision, appropriate training, and seamless integration with the academic curriculum.

Community-oriented medical education programs also play a critical role in promoting social accountability among medical students, preparing them to address the healthcare needs of underserved populations [11]. However, there remains limited evidence evaluating the effectiveness of the FAP using a comprehensive mixed-methods approach that includes perspectives of students, faculty, and community members.

Satna district in Madhya Pradesh represents a region where significant rural-urban disparities in healthcare access and health awareness continue to exist. Many surrounding rural communities face challenges such as limited availability of healthcare facilities, transportation barriers, inadequate health literacy, and dependence on primary healthcare services. These contextual factors implement community-based medical education initiatives such as the FAP, which is particularly relevant, as they provide students with exposure to real-world public health challenges while simultaneously supporting underserved populations.

Accordingly, the present study was conducted with the following objectives: (i) to assess medical students’ perceptions regarding the effectiveness of the FAP in improving clinical, communication, and community-oriented skills; (ii) to explore the experiences and perspectives of adopted families and faculty members regarding the program; and (iii) to identify major challenges, barriers, and opportunities for improving the implementation and sustainability of the FAP.

## Materials and methods

This mixed-methods study was conducted at Government Medical College, Satna, Madhya Pradesh, from January 1, 2025, to March 31, 2025, and comprised both quantitative and qualitative components. A total of 220 participants were included in the study, comprising 150 second-year medical students, 50 adopted family members, and 20 faculty members associated with the FAP.

In the quantitative component, 150 second-year medical students were recruited through convenience sampling. Convenience sampling was used to recruit second-year MBBS students who were actively participating in the FAP during the study period. This approach was adopted considering feasibility, academic schedules, and participant availability. To minimize selection bias and improve representativeness, efforts were made to include students from different tutorial groups and varying demographic backgrounds who had completed sufficient field exposure under the program.

All participants were enrolled in the FAP and had already been assigned to adopted families. Data were collected using a semi-structured, pretested questionnaire consisting of multiple items measured on a 5-point Likert scale ranging from 1 (Never) to 5 (Always). The questionnaire explored students’ experiences, perceptions of the program’s effectiveness, and challenges encountered during implementation. Individual Likert-scale items were treated as ordinal variables and summarized using frequencies and percentages. Medians with interquartile ranges (IQRs) were calculated to describe central tendency. For ease of interpretation, selected response categories were collapsed and presented as “Often/Always” where appropriate. Median Likert scores were calculated for each item to summarize the central tendency of ordinal responses.

The questionnaire was developed after reviewing relevant literature on FAPs and community-based medical education (see the Appendices). Three experts assessed content validity, and necessary revisions were made. It was pilot tested on 15 students outside the study sample to improve clarity. Internal consistency was acceptable, with a Cronbach’s alpha of 0.82.

The qualitative component included 20 faculty members and 50 members of the adopted families. Faculty perspectives were gathered through in-depth interviews (IDIs), while the views of adopted family members were explored through five focus group discussions (FGDs), each conducted in person with 10 participants. 

For the qualitative component, purposive sampling was employed to obtain diverse stakeholder perspectives. Faculty members were selected based on their direct involvement in supervising and coordinating FAP activities. Adopted family members were selected based on regular interaction with students, willingness to participate, and ability to provide detailed feedback regarding their experiences with the program. Participants with varied socioeconomic and educational backgrounds were included to capture a broader range of community perspectives and experiences.

Qualitative data obtained from faculty interviews and FGDs with adopted family members were analyzed using Braun and Clarke’s six-step thematic analysis framework. Initially, audio recordings were transcribed verbatim and reviewed repeatedly to achieve data familiarization. Open coding was then performed independently by two investigators, following which similar codes were grouped into categories and broader themes through iterative discussion and consensus. Themes were reviewed, refined, and finalized to ensure coherence and relevance to the study objectives.

To improve the reliability of qualitative findings, inter-coder agreement was assessed by comparing the consistency of codes assigned independently by the two investigators across selected transcripts. Percentage agreement was calculated by dividing the number of agreed coding instances by the total number of coding decisions and multiplying by 100, which yielded an inter-coder agreement of 92%, indicating high coding consistency and reliability.

Qualitative data were analyzed using Braun and Clarke’s thematic analysis framework with the assistance of NVivo 15 software (Lumivero, Denver, Colorado, USA). After transcription and familiarization, two investigators independently performed open coding of the interviews and FGDs. Similar codes were grouped into categories and subsequently refined into broader themes through iterative discussion and consensus. Themes were reviewed against the original transcripts to ensure credibility and consistency, and representative quotations were used to support the findings.

Ethical approval was obtained from the Institutional Ethics Committee of Government Medical College, Satna, Madhya Pradesh (Ref. No. 139/IEC/GMC/2024) on December 23, 2024. Written informed consent was obtained from all participants. Audio recordings were securely stored as password-protected soft copies under the Principal Investigator's custody. A loosely structured interview guide was used to facilitate discussion while allowing flexibility to probe emerging themes. Reversible coding was used to maintain anonymity, and the same codes were applied to transcripts. No personal or institutional identifiers were included in the final dataset.

Findings from the quantitative and qualitative components were triangulated to provide a comprehensive evaluation of the FAP’s strengths, challenges, and overall impact.

## Results

A total of 220 participants were included in the study, comprising 150 second-year undergraduate medical students, 50 adopted family members, and 20 faculty members from Government Medical College, Satna. Among faculty participants, one was a Professor (including one Head of Department), five Associate Professors, ten Assistant Professors, and four Senior Residents.

Quantitative findings

Perceived Utility of the Family Adoption Program

Most students reported a high perceived educational benefit from participation in the FAP (Table 1). Nearly all respondents indicated that the program enhanced their clinical skills, with 96.7% reporting “often” or “always” experiencing improvement (Median = 5, IQR 4-5). Similarly, 94.0% reported that the program provided meaningful real-world medical exposure (Median = 4, IQR 4-5).

**Table 1:** Perceived Utility of the Family Adoption Program by the Students (n = 150) The median Likert score represents the central tendency of responses on a 5-point Likert scale (1 = Never to 5 = Always) IQR: interquartile range

Item	Never n (%)	Rarely n (%)	Sometimes n (%)	Often n (%)	Always n (%)	Median Likert Score (IQR)
Enhances clinical skills	0 (0.0)	1 (0.7)	4 (2.7)	72 (48.0)	73 (48.7)	5 (4–5)
Provides real-world experience	0 (0.0)	1 (0.7)	8 (5.3)	71 (47.3)	70 (46.7)	4 (4–5)
Supervisor support	0 (0.0)	2 (1.3)	20 (13.3)	75 (50.0)	53 (35.3)	4 (4–5)
Understanding family dynamics	0 (0.0)	1 (0.7)	12 (8.0)	81 (54.0)	56 (37.3)	4 (4–5)
Integration with curriculum	0 (0.0)	3 (2.0)	9 (6.0)	81 (54.0)	57 (38.0)	4 (4–5)

A large proportion of students reported strong supervisory support, with 86% indicating frequent guidance from faculty members (Median = 4, IQR 4-5). Students also perceived improvements in their understanding of family dynamics (91.3%) and integration of community experiences with the academic curriculum (92.0%), suggesting that the program effectively links theoretical learning with community-based practice. Median Likert scores were calculated for each item to provide a summary measure of central tendency alongside categorical response distributions.

Problems Encountered During the Program

Despite positive perceptions, several challenges were identified (Table 2). Nearly half of the students (47.0%) reported difficulty balancing academic workload with program responsibilities. Travel requirements were also a major concern, with 52.3% reporting frequent logistical challenges related to transportation and commuting to adopted villages.

**Table 2:** Problems Faced During the Family Adoption Program (n = 150) The median Likert score represents the central tendency of responses on a 5-point Likert scale (1 = Never to 5 = Always) IQR: interquartile range

Item	Never–Rarely n (%)	Sometimes n (%)	Often–Always n (%)	Median Likert Score(IQR)
Balancing academic workload	21 (13.9)	59 (39.1)	71 (47.0)	4 (3–4)
Travel requirements	27 (17.9)	45 (29.8)	79 (52.3)	4 (3–5)
Communication barriers	33 (21.9)	54 (35.8)	64 (42.4)	3 (3–4)
Cultural differences	37 (24.5)	62 (41.1)	52 (34.4)	3 (3–4)
Interference with personal time	41 (27.2)	49 (32.5)	61 (40.4)	3 (3–4)

Communication barriers were reported by 42.4% of students, reflecting difficulties in language differences, cultural contexts, or explaining medical concepts to community members. Additionally, 40.4% of students reported interference with personal time, highlighting the time-intensive nature of the program. For clearer interpretation, selected response categories for problem-related items were collapsed into broader groups (e.g., “Never-Rarely” and “Often-Always”), a common approach that improves interpretability without obscuring the underlying data.

Overall Experience and Satisfaction

Overall satisfaction with the program was high (Table 3). Approximately 76.8% of students reported being often or always satisfied with the FAP, with a median satisfaction score of 4 (IQR 4-5).

**Table 3:** Overall Experience with the Family Adoption Program (n = 150) The median Likert score represents the central tendency of responses on a 5-point Likert scale (1 = Never to 5 = Always) IQR: interquartile range

Item	≤ Sometimes n (%)	Often n (%)	Always n (%)	Median Likert Score(IQR)
Overall satisfaction	35 (23.2)	76 (50.3)	40 (26.5)	4 (4–5)
Would recommend to peers	13 (8.6)	39 (25.8)	99 (65.6)	5 (4–5)
Likely to continue participation	8 (5.3)	61 (40.4)	82 (54.3)	5 (4–5)

Most students expressed strong endorsement of the initiative, with 91.4% indicating that they would recommend the program to their peers and 94.7% reporting willingness to continue participation. These findings suggest broad acceptance of the program despite operational challenges.

Qualitative findings

For the qualitative aspect of the study, 20 IDIs were conducted with faculty members to gather their insights into the FAP. Additionally, five FGDs were held with adoptive family members, with each group comprising 10 participants. Participation was consistent, and no dropouts occurred during either the interviews or the group discussions. The IDIs lasted between 8 minutes and 54 seconds and 49 minutes and 45 seconds, while the FGDs ranged in duration from 13 minutes and 51 seconds to 45 minutes. Thematic analysis identified four overarching domains consistent with the Strengths, Weaknesses, Opportunities, and Challenges (SWOC) framework.

Faculty Perspectives

Qualitative analysis of IDIs with faculty members indicated that the FAP was widely perceived as beneficial for enhancing students’ communication skills, clinical exposure, empathy, and understanding of social determinants of health. Faculty highlighted that sustained interaction with families helps students link social conditions with clinical problems and develop a more patient-centered approach to care. However, several limitations were noted, including students’ limited clinical experience, logistical issues such as transportation and time constraints, and the challenge of balancing academic workload with field visits. Faculty also identified opportunities for strengthening community engagement, improving health awareness, and using the program as a platform for community-based research, while challenges included communication barriers, safety concerns during village visits, and the need for better training and mentorship (Table 4).

**Table 4:** Faculty Perspectives with the Family Adoption Program (n=20) NMC: National Medical Commission

Category	Theme	Sub-theme	Faculty	Exact Statement
Strengths	Skill Development	Communication and clinical skills	F1	“The FAP will improve their communication as well as clinical skills as they have gained exposure to clinical skills in the 2nd year. At that time, they had already developed their communication skills, so they could easily correlate the social problems with clinical issues and make clinic-social diagnosis.”
	Learning Interest	Making study interesting	F1	“The FAP can make studying diseases and health problems much more interesting.”
	Knowledge Enhancement	Experience and learning	F3	“Participating in the scheme will boost their knowledge and skills, providing valuable experience by the end of three years.”
	Doctor–Patient Interaction	Counselling and communication	F5	“The program enhances communication skills by teaching students to interact, counsel, and assure patients, while benefiting society through early diagnosis, education, and doctor-patient connections.”
	Empathy & Social Responsibility	Long-term family engagement	F5	“From the start, students will engage with a family for community participation, learning to care for them as their own. Over five years, they’ll realize the impact they made—helping the family medically and socially, fostering empathy and fulfilment.”
	Community Engagement	Rural healthcare connection	F6	"By connecting students with rural communities, this program enhances healthcare access, fosters student growth, and builds positive relationships between the medical college and the community."
Weaknesses	Health Advice Limitations	Practical applicability	F1	“If the health advice provided by students is not practical or relevant to the specific needs and circumstances of the families, they may not find it useful or helpful.”
	Information Accuracy	Risk of misinformation	F1	“If students provide inaccurate or incomplete health information, it can lead to confusion, frustration, and mistrust among families.”
	Academic Burden	Additional workload	F2	“Students considered it as an extra burden on them by the NMC.”
	Logistical Issues	Transport and commuting	F4	“Students may face commuting issues, lack of transport, and bad weather. During exams, they might feel their time is wasted, leading to a lack of proper involvement. Additionally, allocating five families per student is impractical in villages with fewer families.”
	Time Constraints	Whole day visits	F6	“One visit takes up the whole day, making it tough for students to continue with classes. A separate time frame for the program would help.”
	Healthcare System Limitations	Dissatisfaction with services	F8	"Families may lose interest in the FAP if they experience inadequate or untimely care at associated health facilities, encounter long wait times for appointments or treatments, or feel that their cultural and social needs are not adequately addressed."
	Social Fear	Fear of authority intervention	F9	“Families may fear that their involvement in outreach programs will lead to unwanted intervention from social services or other authorities. This fear can prevent them from seeking help and participating in programs that could benefit them.”
	Social Stigma	Family structure discrimination	F9	"Families with complex structures, such as single-parent households, blended families, or families with LGBTQ+ members, may face stigma and discrimination within their communities. This can make them hesitant to open up to outsiders and participate in outreach programs.”
Opportunities	Community Understanding	Social determinants of health	F1	“The students will get to know about the major social challenges and community problems, and also understand the gravity of the problems from the very start of their course duration.”
	Health Awareness	Chronic disease awareness	F2	“The families will be much more aware of their chronic illness and health problems running in the family.”
	Health-seeking Behaviour	Knowledge of healthcare services	F2	“By this programme, the adopted families are aware of the illness symptoms, where and whom they have to reach to avail medical facilities.”
	Academic Motivation	Interest in disease learning	F2	“FAP will increase students’ eagerness about learning new diseases, how to screen, diagnose, and manage the patients.”
	Experiential Learning	Hands-on training	F3	“Over the three years, this program will enrich their knowledge and prepare them with hands-on experience.”
	Communication Skills	Observation and interaction	F4	“Such programs enhance students' communication and observational abilities through exposure to diverse patients.”
	Program Development	Monitoring and communication	F6	“To improve our Family Adoption Program, we need to track student progress, assess student needs, and enhance administrative communication.”
	Community Collaboration	Partnerships with local organizations	F8	“By collaborating with community organizations that have established trust within vulnerable populations. This can help to build bridges with families and facilitate their participation in outreach programs.”
	Research Potential	Community health research	F9	“FAP can serve as a platform for research and innovation in community health, allowing students and faculty to investigate local health issues and develop innovative solutions.”
Challenges	Academic Stress	Managing studies and FAP	F1	“Students may feel stressed as they have to manage the family adoption program alongside three other subjects. They also face transportation issues, unfavourable weather, and are often taken for visits during exam prep time, leading to disinterest despite having to attend.”
	Communication Barriers	Convincing families	F2	“Students need guidance on communication and persuading families to join, as not all families will agree. Language barriers also pose a challenge for non-local students.”
	Social Stigma	Mental illness and addiction	F3	“Families dealing with health issues like mental illness, substance abuse, or chronic diseases might face stigma and discrimination within their communities. This can make them hesitant to engage with outsiders, including student volunteers.”
	Safety Issues	Transport and security	F3	“The main challenges include transport and safety, especially for female students and faculty, as well as food arrangements during village visits. Proper guidance and teaching in class are essential to prepare students for the problems they may face when engaging with families in the village.”
	Security Risk	Criminal history families	F4	“The program is well-intentioned, but resistance from families with criminal histories could create potential risks for the students.”
	Long-term Impact	Delayed program outcomes	F5	“The program's effectiveness will show over time. While first-year students' impact is uncertain now, it will benefit families by increasing awareness of medical health and facilities.”
	Privacy Concerns	Fear of judgement	F5	“Families may fear that their health issues will be judged or that their privacy will be violated. This can create a barrier to open communication and trust-building.”
	Program Design	Managing multiple families	F6	“The family adoption program is a great idea, but it's challenging for first-year students to manage five families at once since their knowledge and thought process differ from those of third-year students. It’s better to start with one or two families and gradually increase the number.”
	Resource Limitations	Limited healthcare access	F6	“Families facing health challenges may already have limited access to healthcare resources. They might be wary of engaging with students, fearing that this will not lead to meaningful support or improvement in their situation.”
	Health Education	Vaccine myths and nutrition counselling	F7	“Students face challenges like building trust, addressing vaccine myths, and counselling families on common nutritional issues.”
	Training Needs	Communication and empathy training	F8	“Training must go beyond formats to include communication, empathy, and diagnosis skills, supported by seniors for better learning and interaction.”
	Socioeconomic Stigma	Poverty-related discrimination	F9	“Families from low-income backgrounds or those facing poverty might encounter stigma and judgment due to their socioeconomic status. This can create feelings of shame and embarrassment, making them less likely to engage with outreach programs.”

Adopted Family Members’ Perspectives

FGDs with adopted family members revealed generally positive perceptions of the program. Families appreciated the students’ friendly approach, home visits, and the health education provided on hygiene, disease prevention, and lifestyle practices. Participants reported increased awareness about health problems and government health schemes. However, some families found medical terminology difficult to understand and noted irregularity in visits. A few participants also expressed uncertainty about relying on advice from students who were still in training. Despite these concerns, the program was perceived as helpful in improving health awareness and encouraging better health-seeking behavior (Table 5).

**Table 5:** Perceptions of Adopted Family Members Regarding the Family Adoption Program (SWOC Analysis) (n=50) SWOC: Strengths, Weaknesses, Opportunities, and Challenges

Theme	Sub-theme	Illustrative Quote
Strengths	Good communication	“They explained things well and seemed genuinely interested in helping us.”
	Approachability	“I can talk about anything with them without feeling awkward.”
	Health education	“They taught us many things about health that we did not know earlier.”
	Home visits	“It was helpful when they checked our homes and advised us about cleanliness.”
Weaknesses	Medical terminology	“Sometimes it is hard to understand their words.”
	Irregular visits	“We never know when they are coming.”
	Limited services	“If you have something serious, you still have to go to the hospital.”
Opportunities	Health awareness	“We are now more aware of health problems.”
	Scheme awareness	“We learned about government health schemes.”
	Behavior change	“Advice on quitting smoking and drinking was helpful.”
Challenges	Trust concerns	“They know some things, but they are not doctors.”
	Practical barriers	“Some advice is difficult to follow in our daily lives.”
	Availability issues	“Family members are not always at home when they visit.”

**Figure 1:**
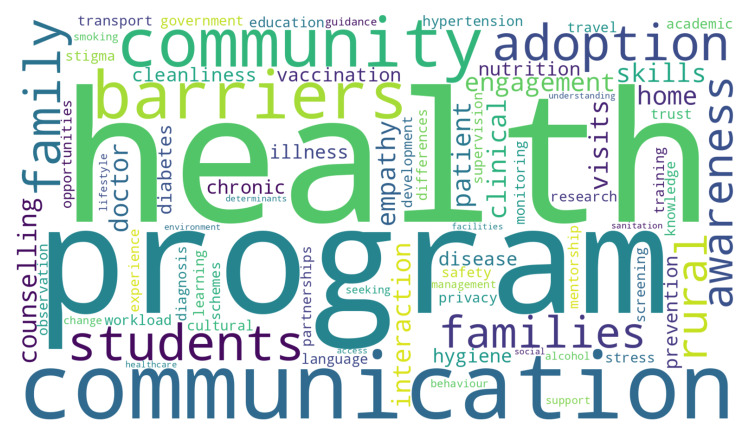
Word Cloud Illustrating Frequently Occurring Themes From Qualitative Interviews and Focus Group Discussions in the Family Adoption Program Study. The word cloud represents commonly used terms from qualitative responses of faculty members and adopted family participants. Words appearing in larger size indicate higher frequency and greater prominence in stakeholder perceptions of the Family Adoption Program

Figure 1 depicts the word cloud of participants’ responses, with frequently occurring words such as “students,” “communication,” “families,” and “conditions.” This indicates that the FAP was mainly perceived in terms of student involvement, family interaction, and communication-related health experiences.

## Discussion

The findings of this study indicate that the FAP is perceived as a valuable educational intervention that enhances community-oriented medical training while simultaneously improving health awareness among adopted families. Students reported improvements in communication skills, clinical reasoning, understanding of social determinants of health, and real-world clinical exposure, while faculty members highlighted its role in bridging theoretical knowledge with practical application. These findings align with the principles of CBME described by Frank et al. [2], which emphasize experiential learning and the development of practical competencies through real-world exposure.

The observed benefits of the program are further supported by the systematic review conducted by Dornan et al. [3], which highlighted the importance of early clinical and community exposure in enhancing learning outcomes among medical students. Students in the present study also demonstrated increased awareness of social determinants of health, a finding consistent with Marmot et al. [4], who emphasized the critical role of social and environmental factors in shaping health outcomes and healthcare access. Furthermore, long-term engagement with families appeared to promote empathy, patient-centered care, and problem-solving abilities, supporting the experiential learning theory proposed by Kolb [5], which suggests that learning is strengthened through direct experience, reflection, and application.

The positive educational impact observed in the present study is comparable to findings reported by Reshmi et al. [6], who found that participation in the FAP enhanced students’ community orientation and practical learning experiences. Similar observations were reported by Ganganahalli et al. [7], who demonstrated that FAP significantly improves students’ understanding of community health dynamics and healthcare delivery. Collectively, these studies reinforce the value of community-based learning as an integral component of undergraduate medical education.

Communication skill development emerged as one of the major benefits reported by students. This finding is supported by Silverman et al. [8], who highlighted the importance of structured communication training in improving patient understanding and satisfaction. Effective communication becomes particularly important in community settings where cultural beliefs, literacy levels, and health perceptions may vary substantially. Trust-related concerns expressed by some adopted families further emphasize the importance of relationship-building within community-based educational programs. Hall et al. [9] demonstrated that patient trust significantly influences engagement with healthcare services and adherence to medical advice. Limited confidence in students’ clinical abilities may therefore reduce participation and acceptance of healthcare recommendations, highlighting the importance of faculty supervision and continuity of interactions.

Despite the overall positive perceptions, several operational challenges were identified. A substantial proportion of students reported difficulty balancing academic workload with FAP responsibilities. These findings are consistent with those reported by Arumugam et al. [10], who identified academic burden, transportation difficulties, and logistical constraints as significant barriers to successful program implementation. Such challenges suggest the need for improved institutional support and better alignment of community activities with academic schedules.

The broader significance of the FAP extends beyond educational outcomes and supports the concept of social accountability in medical education proposed by Boelen and Heck [11]. By exposing students to real-world community health needs, the program contributes to the development of socially responsible healthcare professionals capable of addressing health inequities and underserved populations. The perspectives of adopted families in the present study further demonstrated positive community-level impacts, including improved awareness of hygiene practices, disease prevention, and available healthcare services. Similar findings have been reported by Eftekhari et al. [12], who observed that community-based programs can improve health literacy and promote preventive healthcare behaviors, particularly among underserved communities.

Communication barriers and cultural differences nevertheless remained important challenges. Students often reported difficulty conveying health information in a manner that was understandable and acceptable to community members. Similar observations have been reported by Tanwani et al. [13], who emphasized the importance of structured communication training for effective doctor-patient interactions. The qualitative component of the present study, analyzed using thematic analysis based on the framework proposed by Braun and Clarke [14], provided valuable insights into these contextual challenges and enabled a deeper understanding of stakeholder experiences.

Another important finding was the potential of the FAP as a platform for community-based research and public health interventions. Faculty participants emphasized its utility in identifying local health issues and generating context-specific solutions. Similar observations have been reported by Yalamanchili et al. [15], who highlighted the role of FAP in strengthening community engagement and promoting public health research. Additional studies by Sulgodu Ramachandra et al. [16], Kumar et al. [17], and Sharma et al. [18] have also underscored the importance of communication skills, community engagement, and structured mentorship in maximizing the effectiveness of community-based medical education programs.

The findings of this study have important implications for medical education, practice, and policy. Strengthening the FAP through enhanced communication training, structured orientation sessions, faculty mentorship, and logistical support may improve both educational outcomes and community engagement. Programs such as FAP therefore have significant potential to contribute to the development of socially accountable and community-oriented healthcare professionals.

A major strength of the present study was its mixed-methods design, which enabled a comprehensive evaluation of the FAP from the perspectives of students, faculty members, and adopted families. The integration of quantitative and qualitative findings provided a more holistic understanding of both educational benefits and implementation challenges. However, several limitations should be acknowledged. The study was conducted at a single institution and utilized convenience sampling for the quantitative component, which may limit the generalizability of the findings. The reliance on self-reported perceptions introduces the possibility of response bias and social desirability bias, whereby students, faculty members, and adopted families may have over-reported favorable experiences or responses due to perceived academic, professional, or institutional expectations despite assurances of confidentiality. In addition, the cross-sectional design precluded assessment of long-term outcomes, and objective measures of skill acquisition were not evaluated.

Future multicenter and longitudinal studies are warranted to assess the sustained impact of the FAP on student competencies, professional development, and community health outcomes. Further research incorporating objective assessments of clinical and communication skills, as well as subgroup analyses across different demographic and community settings, may provide deeper insights into factors influencing program effectiveness and help optimize its implementation across diverse healthcare contexts.

## Conclusions

The FAP is an effective community-based learning strategy that enhances medical students’ communication skills, clinical understanding, and awareness of social determinants of health while promoting health awareness among adopted families. Although participants reported substantial educational and community benefits, challenges related to logistics, academic workload, and communication barriers remain. Strengthening institutional support through structured mentorship, adequate logistical resources, and better integration of the program within the medical curriculum may improve its effectiveness and sustainability. These findings support the incorporation of well-structured community-engagement programs as an integral component of CBME.

## Appendices

Appendix 1: faculty interview guide

In-Depth Interview Guide for Faculty Members: Perceptions of the Family Adoption Program

Introduction

Please briefly describe your role and involvement in the Family Adoption Program.

General Perceptions of the Program

What are your overall thoughts and perceptions regarding the Family Adoption Program?

Impact on Students

In your opinion, how does participation in the Family Adoption Program influence the academic performance of medical students? Have you noticed any perceived changes in students’ clinical skills following participation in the program? How has the program influenced students’ communication skills with patients and families? Have you observed any changes in students’ social or emotional development as a result of participation in the program? Please share any specific examples or observations, if applicable.

Program Utility and Educational Value

How do you think the Family Adoption Program contributes to the medical education curriculum? Which aspects of the program do you believe are most effective? What skills or competencies do students appear to gain through participation in the program?

Challenges and Barriers

What challenges do students commonly face while participating in the program? Have you observed any academic, logistical, or personal difficulties among students? Do you experience any challenges as a faculty member while supervising or supporting students in the program? What type of support do you think is necessary for students and faculty members involved in the program?

Program Structure and Support System

How well structured and organized do you feel the program is? What areas of the program require improvement? What type of orientation or preparation is provided to students before participation? Do you consider this preparation adequate? Please explain your perspective. How effective is the existing support system for students during program implementation? What additional resources or support mechanisms would you recommend?

Impact on Faculty Members

How has the Family Adoption Program influenced your workload and professional responsibilities? What benefits or difficulties have you personally experienced while participating in the program?

Overall Evaluation and Recommendations

Overall, how satisfied are you with the Family Adoption Program? Which aspects of the program function effectively? Which aspects require improvement? What recommendations would you suggest for strengthening the Family Adoption Program? How can the program better support students, faculty members, and adopted families?

Closing Question

Is there anything else you would like to share regarding your experience or perceptions of the Family Adoption Program?

Appendix 2: student questionnaire

Evaluation of the Family Adoption Program Among Medical Students

Response Scale

The following response scale was used for Questions 3-20: Always | Often | Sometimes | Rarely | Never

Section A: General Information

Question 1

What is your current year of medical study? 1st Year 2nd Year 3rd Year 4th Year Final Year Internship

Question 2

How long have you been participating in the Family Adoption Program? Less than 6 months 6 months-1 year 1-2 years 2-3 years More than 3 years

Section B: Utility of the Family Adoption Program

Question 3

How often does the Family Adoption Program help enhance your clinical skills?

Question 4

How often does the program provide meaningful real-world medical exposure?

Question 5

How often has the program helped improve your communication skills with patients?

Question 6

How often does the program improve your understanding of family dynamics and their influence on health?

Question 7

How often is the program effectively integrated with the medical education curriculum?

Section C: Challenges Faced During Participation

Question 8

How often do you experience difficulty balancing program responsibilities with academic workload?

Question 9

How often do travel requirements make family visits difficult?

Question 10

How often do you feel adequately supported by supervisors during the program?

Question 11

How often do you encounter communication barriers while interacting with families?

Question 12

How often do cultural differences create challenges during family interactions?

Question 13

How often do you feel the pre-program orientation or training was adequate for your role?

Question 14

How often does participation in the program interfere with your personal time or well-being?

Section D: Overall Experience

Question 15

How often are you satisfied with your experience in the Family Adoption Program?

Question 16

How often would you recommend that other medical students participate in the program?

Question 17

How often do you feel motivated to continue participating in the program?

Section E: Additional Feedback

Question 18

How often do you perceive significant benefits from participation in the program?

Question 19

How often do you encounter major challenges while participating in the program?

Question 20

How often do you feel that improvements are needed in the program?

Appendix 3: focus group discussion guide

Experiences and Perceptions of Adopted Family Members Regarding the Family Adoption Program

Section 1: Initial Experiences

Please describe your first interaction with the medical students when they approached your family for participation in the program. What were your initial thoughts or feelings about being included in the Family Adoption Program? How comfortable did you and your family feel during the initial visits?

Section 2: Overall Experience and Participation Journey

How has your overall experience with the Family Adoption Program been so far? In what ways has the program influenced your family’s health awareness, lifestyle, or daily routine? Which aspects of the program did you find most beneficial or helpful? What challenges or difficulties did you experience while participating in the program? What type of support, guidance, or resources provided by the students were most useful to your family?

Section 3: Health Services and Medical Support

Please share your experience regarding the health-related services or support provided by the medical students. Did you or any family member receive medical advice, health check-ups, or healthcare guidance from the students? If yes, how helpful was it? Did you face any difficulty accessing or using the healthcare services recommended by the students? Were referrals made to health centers or hospitals when required? If yes, please describe your experience.

Section 4: Health Education and Awareness

Did the students provide health education, awareness sessions, or health-related information to your family? Which health-related topics were discussed during the visits, such as hygiene, nutrition, maternal and child health, or chronic disease prevention? How useful was this information for you and your family members? Was there any additional information, training, or support you feel should have been provided?

Section 5: Communication and Relationship Building

How would you describe your interaction and communication with the medical students during the program? Did you feel comfortable discussing your family’s health concerns with the students? Please explain your experience. Did the students listen carefully to your concerns and respond appropriately?

Section 6: Closing Questions

Overall, how satisfied are you with your experience in the Family Adoption Program? What changes or improvements would you suggest to make the program more beneficial for families? Is there anything else you would like to share regarding your experience with the program?
